# Functions of BCL-X_**L**_ at the Interface between Cell Death and Metabolism

**DOI:** 10.1155/2013/705294

**Published:** 2013-02-28

**Authors:** Judith Michels, Oliver Kepp, Laura Senovilla, Delphine Lissa, Maria Castedo, Guido Kroemer, Lorenzo Galluzzi

**Affiliations:** ^1^INSERM, U848, Institut Gustave Roussy, Pavillon de Recherche 1, 39 Rue Camille Desmoulins, 94805 Villejuif, France; ^2^Université Paris Sud/Paris XI, 94805 Villejuif, France; ^3^Institut Gustave Roussy, 94805 Villejuif, France; ^4^Université Paris Descartes/Paris V, Sorbonne Paris Cité, 75006 Paris, France; ^5^Metabolomics Platform, Institut Gustave Roussy, 94805 Villejuif, France; ^6^Pôle de Biologie, Hôpital Européen Georges Pompidou, AP-HP, 75015 Paris, France; ^7^Equipe 11 Labelisée par la Ligue Nationale Contre le Cancer, Centre de Recherche des Cordeliers, 75006 Paris, France

## Abstract

The BCL-2 homolog BCL-X_L_, one of the two protein products of *BCL2L1*, has originally been characterized for its prominent prosurvival functions. Similar to BCL-2, BCL-X_L_ binds to its multidomain proapoptotic counterparts BAX and BAK, hence preventing the formation of lethal pores in the mitochondrial outer membrane, as well as to multiple BH3-only proteins, thus interrupting apical proapoptotic signals. In addition, BCL-X_L_ has been suggested to exert cytoprotective functions by sequestering a cytosolic pool of the pro-apoptotic transcription factor p53 and by binding to the voltage-dependent anion channel 1 (VDAC1), thereby inhibiting the so-called mitochondrial permeability transition (MPT). Thus, BCL-X_L_ appears to play a prominent role in the regulation of multiple distinct types of cell death, including apoptosis and regulated necrosis. More recently, great attention has been given to the cell death-unrelated functions of BCL-2-like proteins. In particular, BCL-X_L_ has been shown to modulate a number of pathophysiological processes, including—but not limited to—mitochondrial ATP synthesis, protein acetylation, autophagy and mitosis. In this short review article, we will discuss the functions of BCL-X_L_ at the interface between cell death and metabolism.

## 1. Introduction

According to current models, cell death most often proceeds via either of two relatively independent subroutines, apoptosis, and necrosis [1, 2]. For a long time, apoptotic and necrotic instances of cell death have exclusively been identified based on morphological criteria [2]. In addition, while apoptosis was believed to constitute the sole regulated (i.e., genetically encoded, and hence susceptible to pharmacological modulation) modality of cell death, necrosis was viewed as a purely accidental process [3]. Recently, a functional classification of cell death mechanisms, based on measurable biochemical features, has been proposed [1], and the concept of regulated necrosis has gained large consensus [4]. In this scenario, the true relevance of additional processes that were previously catalogued as *bona fide* cell death subroutines is being reevaluated. In particular, while macroautophagy (hereafter referred to as autophagy) turned out to constitute a prominent homeostatic and cytoprotective mechanism [5, 6], autophagic cell death (a lethal subroutine that is mediated, rather than merely accompanied, by autophagy) has been shown to occur in a limited number of, mostly developmental, scenarios [1, 7]. Along similar lines, mitotic catastrophe, a signaling cascade elicited in mitosis-incompetent cells that was initially viewed as a particular case of apoptosis [8], has recently been proposed to constitute an oncosuppressive mechanism with multiple functional outcomes, including cell senescence as well as apoptotic and necrotic cell death [9].

 Apoptotic stimuli can be propagated via two distinct, but not entirely disjointed, molecular cascades: extrinsic apoptosis, transducing lethal signals that originate in the extracellular microenvironment, and intrinsic (also known as mitochondrial) apoptosis, responding to perturbations of intracellular homeostasis [10]. Extrinsic apoptosis can be initiated either by the ligand-induced activation of plasma membrane death receptors (e.g., FAS/CD95, tumor necrosis factor receptor 1 (TNFR1)) or by so-called dependence receptors (e.g., deleted in colorectal carcinoma (DCC)), when the concentration of their ligands falls below a specific threshold [1]. Death receptors promote the activation of caspases (a class of cysteine proteases that play a central role in multiple instances of apoptosis) [11] via the formation of a multiprotein complex that includes—among other components—receptor-interacting protein kinase 1 (RIPK1), FAS-associated protein with death domain (FADD), cellular inhibitor of apoptosis proteins (cIAPs), and multiple isoforms of cellular FLICE-inhibitory protein (c-FLIP). Such a death-inducing signaling complex (DISC) allows for the proximity-induced autoactivation of caspase-8, in turn catalyzing the proteolytic maturation of caspase-3, the central effector of most cases of apoptosis [10]. The mechanisms whereby dependence receptors are connected to the execution of apoptosis have only recently begun to emerge and appear to involve caspase-9, a caspase that was long believed to exclusively regulate mitochondrial apoptosis [12].

Intrinsic apoptosis can be triggered by a plethora of perturbations in intracellular homeostasis, including—among others—DNA damage, oxidative stress, and cytosolic Ca^2+^ overload [10]. Independent of the initiating stimulus, the signaling cascades that mediate intrinsic apoptosis as well as the prosurvival signals that are generated alongside (to facilitate the reestablishment of homeostasis) are opposed to each other at the level of mitochondria [13–15]. If lethal signals prevail, the majority of mitochondria become permeabilized, an event that *de facto* seals the cell fate. Indeed, upon mitochondrial outer membrane permeabilization (MOMP), (i) the mitochondrial transmembrane potential (Δ*ψ*_*m*_), that is, the electrochemical gradient driving ATP synthesis as well as many other mitochondrial functions, is rapidly dissipated; and (ii) cytotoxic proteins that are normally secluded within the mitochondrial intermembrane space (e.g., cytochrome c; apoptosis-inducing factor (AIF), endonuclease G (ENDOG)) are released into the cytosol, where they promote the activation of caspases as well as of caspase-independent cell death executioner mechanisms. The former relies on the cytochrome *c*-elicited, dATP-, and apoptotic peptidase activating factor 1 (APAF1)-dependent assembly of the so-called apoptosome, a molecular platform for the activation of the caspase-9 → caspase-3 cascade. The latter involves the caspase-independent endonuclease activity of AIF and ENDOG as well as the bioenergetic and redox crisis that ensues Δ*ψ*_*m*_ dissipation [13, 14]. Of note, extrinsic and intrinsic apoptosis are not entirely disjointed. Indeed, while in some cell types (e.g., lymphocytes) the caspase-8 → caspase-3 cascade is sufficient to mediate death receptor-dependent apoptosis, in others (e.g., hepatocytes), this process requires the caspase-8-mediated cleavage of the BH3-only protein BID, generating a mitochondrion-permeabilizing fragment (see below) [16, 17].

Given its position at the frontier between cell life and death, it is not surprising that MOMP constitutes a highly regulated phenomenon. So far, two models have been put forward to explain MOMP in molecular terms [14, 18]. On one hand, MOMP has been suggested to originate at the mitochondrial outer membrane (OM), thanks to the pore-forming activity of multidomain proapoptotic members of the BCL-2 protein family, namely, BAX and BAK [14, 19]. On the other hand, it has been proposed that—in response to specific triggers—MOMP would stem from the so-called mitochondrial permeability transition (MPT), an abrupt increase in the permeability to small solutes of the mitochondrial inner membrane (IM). In this latter scenario, a critical role has been ascribed to the permeability transition pore complex (PTPC), a large molecular entity assembled at the junctions between the OM and the IM by several proteins, including (though presumably not limited to) voltage-dependent anion channels (VDACs), adenine nucleotide translocase (ANTs), and cyclophilin D (CYPD) [13, 18].

Importantly, antiapoptotic multidomain members of the BCL-2 protein family, including BCL-2 itself, BCL-X_L_, and MCL-1, not only counteract the pore-forming activity of BAX and BAK by engaging in direct inhibitory interactions, but also (i) intercept upstream proapoptotic signals such as those mediated by BH3 only proteins like BAD, BID, BIM, and BBCR3 (best known as p53-upregulated modulator of apoptosis (PUMA)) [20, 21], (ii) bind to, hence regulating, several components of the PTPC, including VDAC1 and ANT [22–24], and (iii) prevent the generation of proapoptotic cytosolic Ca^2+^ waves, either by interacting with inositol 1,4,5-trisphosphate (IP3)-gated Ca^2+^ channels on the endoplasmic reticulum (ER) or by limiting the capacity of ER Ca^2+^ stores [25–27]. In addition, both pro- and antiapoptotic BCL-2-like proteins have recently been shown to modulate multiple processes that are not directly connected to the execution of cell death, including—among others—bioenergetic metabolism, mitochondrial functions, mitosis, and autophagy [28, 29].

Here, we discuss the multifaceted role of BCL-X_L_, a prototypic antiapoptotic member of the BCL-2 family, at the hub between cell death and metabolism.

## 2. BCL-X_**L**_ and Cell Death

In humans, BCL-X_L_ is encoded by *BCL2L1*, a *BCL2*-related gene mapping to chromosome 20q11.21 [30]. *BCL2L1* was shown from the beginning to code for two distinct protein products, owing to the alternative splicing of *BCL2L1* mRNA: a cytoprotective factor of 233 residues (BCL-X_L_) and a smaller polypeptide (170 residues) that exerts BCL-X_L_-antagonizing functions (BCL-X_S_) [30]. Similar to BCL-2, BCL-X_L_ contains four distinct BCL-2 homology (BH) domains (BH1–BH4) as well as a transmembrane region, through which it localizes—at least in part—to several membranous compartments, including the OM, the ER, and the nuclear envelope [30–32] The same does not apply to BCL-X_S_, which lacks both the BH1 and BH2 domains [30, 33]. Of note, in most physiological settings the BCL-X_L_-coding mRNA is expressed to higher levels than its BCL-X_S_-coding counterpart [34]. Conversely, BCL-X_S_ often predominates in situations of developmental and pharmacological cell death [35, 36]. Importantly, a caspase-generated cleavage product of BCL-X_L_ (lacking an N-terminal fragment) has recently been shown to mediate neuronal cell death in rodent models of ischemic brain injury [37], suggesting that chemical inhibitors of BCL-2-like proteins might also be employed (at least in selected circumstances) as cytoprotective agents.

BCL-2 and BCL-X_L_ were soon recognized as critical antiapoptotic factors, although this function was initially attributed to their ability to mediate antioxidant effects [38, 39]. This notion has quickly been abandoned in favor of the so-called “rheostat” model, proposing that BCL-2 and BCL-X_L_ would physically sequester their proapoptotic counterparts BAX and BAK in inhibitory interactions [40–42]. In the following decade, along with the discovery of several other members of the BCL-2 protein family, this model has been progressively refined to include the concepts of “activating” and “derepressing” BH3-only proteins [21]. According to current viewpoints, the former would promote MOMP by engaging in direct activatory liaisons with BAX and BAK, while the latter would do so by competitively displacing BAX and BAK from inhibitory interactions with BCL-2, BCL-X_L_, and MCL-1 [21]. Of note, the core concept of the rheostat model as first theorized by Stanley Korsmeyer in 1993 [43], that is, that cell death is governed by the balance between pro- and antiapoptotic BCL-2 family members, has remained remarkably unmodified since its original formulation.

Nevertheless, during the last two decades, antiapoptotic BCL-2 family members, including BCL-X_L_, have been shown to exert cytoprotective functions via a myriad of mechanisms that do not necessarily rely on their capacity to block the pore-forming activity of BAX and BAK although—at least in some instances—they do involve a BAX-/BAK-antagonizing effect. Similar to BCL-2, BCL-X_L_ prevents the generation of proapoptotic cytosolic Ca^2+^ waves by reducing capacity of ER Ca^2+^ stores, an effect that is antagonized by BAX and BAK [25–27]. Moreover, BCL-X_L_ has been shown to critically regulate the opening status of VDAC1, and hence of the PTPC, thus influencing MPT-dependent apoptotic cell death [23, 24, 44].

Of note, while some authors proposed that the MPT would stem from an unselectively open conformation of the PTPC [23], others concluded that the MPT would originate from the closed state of the pore [24, 44]. Irrespective of this controversy, which has not yet been fully resolved, recent data have confirmed a critical role for the interaction between VDAC1 and BCL-X_L_ in the antiapoptotic properties of the latter [45]. Interestingly, many other BCL-2 family members such as BAX, BAK, BID, and BCL-2 appear to interact with (and hence modulate the activity of) PTPC components (i.e., VDAC1, VDAC2, and ANT) [22, 23, 46, 47], suggesting that the crosstalk between these two systems might constitute a particularly important point of functional regulation. However, the actual relevance of the PTPC for the cellular demise in physiological settings remains matter of debate. Indeed, mice lacking one or more of the most critical PTPC components (including all VDAC and ANT isoforms known thus far) [48–50], with the single exception of *Ppif* ^−/−^ animals (lacking CYPD) [51–53], fail to exhibit remarkable cell death defects in response to ischemic, traumatic, and pharmacological challenges. Hence, it seems that—at least in physiological settings—the complex crosstalk between BCL-2 family members and the PTPC mainly modulates cell-death unrelated cellular functions and impacts on the cellular demise only via indirect circuitries (see below).

One of the most central regulators of apoptosis as triggered by perturbations of intracellular homeostasis such as DNA damaging conditions, imbalances in redox homeostasis and oncogenic stress is p53 [54]. Besides operating as a stress-responsive transcription factor that regulates the synthesis of both pro- and antiapoptotic proteins, including a large panel of BCL-2 family members (i.e., BAX, BAK, BAD, BID, PUMA, BCL-2, and BCL-X_L_) [55], p53 can also exert apoptotic functions in a transcription-independent fashion [56, 57]. In particular, p53 has been shown to operate similar to BH3-only proteins, that is, to promote MOMP either by engaging in activatory (though labile) interactions with BAX [58] or by displacing BAX and BAK from inhibitory liaisons with BCL-2 and BCL-X_L_ [59]. In this setting, the mitochondrial pools of BCL-2 and BCL-X_L_ constitute the main target for the “derepressor” activity of p53 [59]. In addition, a cytoplasmic pool of BCL-X_L_ appears to work as a PUMA-sensitive inhibitor of p53, *de facto* operating at the interface between p53 transcriptional and transcription-independent functions [60]. Thus, BCL-X_L_ exerts cytoprotective effects not only as it antagonizes its proapoptotic counterparts but also as it counteracts the activity of p53. In addition, BCL-X_L_ has recently been reported to interact with the mitochondrial phosphatase phosphoglycerate mutase family member 5 (PGAM5) [61], a central effector of regulated necrosis [62, 63]. Hence, although Niture et al. did not address this question in a direct fashion, BCL-X_L_ may soon be discovered to regulate necrotic instances of cell death.

## 3. BCL-X_**L**_ and Metabolism

The hypothesis that BCL-2 family members, notably BAX, BAD, BCL-2, and BCL-X_L_, would influence bioenergetic and intermediate metabolism began to gain consensus along with the discoveries that (i) these proteins interact with components of the PTPCs that, in physiological circumstances, regulate various facets of mitochondrial functions (e.g., ANT, VDAC, and glucokinase) [22–24, 44, 64], (ii) these proteins modulate Ca^2+^ homeostasis at the ER [25–27], and (iii) p53 not only operates as a potent proapoptotic factor in response to stress but also exerts an homeostatic control over metabolism [54]. In particular, BCL-X_L_ (i) reportedly preserves the physiological conformation of VDAC, hence promoting the exchange of metabolites, including ADP, across the OM [44]; (ii) functionally antagonizes BAD [42], which has been found to exert prominent metabolic functions by regulating a mitochondrial multiprotein complex that involves (among other enzymes) glucokinase, protein kinase A, and protein phosphatase 1 [64]; (iii) has been shown to lower the concentrations of Ca^2+^ ions within the ER, hence quenching the bioenergetic burst that normally results from the opening of IP_3_-gated Ca^2+^ channels [25–27]; and (iv) binds to cytoplasmic p53 in steady-state conditions [60], thus—at least theoretically—modulating its functions related to bioenergetic and redox metabolism [54, 65, 66].

BCL-X_L_ has also been demonstrated to regulate distinct facets of intermediate metabolism in a direct fashion. In neurons, a pool of BCL-X_L_ localized to the IM appears to physically interact with the *β*  subunit of the F_1_F_O_ ATP synthase, hence increasing its enzymatic efficiency, stabilizing the Δ*ψ*_*m*_ and—consequently—maximizing mitochondrial ATP generation [67, 68]. Similar functions have been attributed to a truncated variant of MCL-1 that localizes to the mitochondrial matrix [69]. In addition, the transfection-enforced overexpression of BCL-2 has been associated with increased oxygen consumption and higher rates of mitochondrial respiration [70, 71]. Taken together, these observations suggest a conserved role for BCL-2 proteins in the regulation of ATP synthesis. Of note, antiapoptotic members of the BCL-2 family have been suggested to exert prooxidant functions, at least under selected circumstances [70, 71]. Such a (slight) prooxidant state, presumably reflecting the ability of BCL-2 and BCL-X_L_ to stimulate mitochondrial respiration [67, 68, 70], appears to be linked to the interaction of BCL-2-like proteins with small GTPases of the RAC family [72, 73] and to exert cytoprotective effects by contributing to the maintenance of baseline energetic homeostasis [71].

Recent data have indicated that BCL-X_L_ operates (in a BAX- and BAK-independent manner) to limit the intracellular levels of acetyl-CoA [74]. Acetyl-CoA is not only a critical intermediate of the Krebs cycle, but also required for protein acetylation, including N-*α*-acetylation, that is, the posttranslational modification that consists in the addition of an acetyl moiety (provided by acetyl-CoA) to the N-terminus of nascent polypeptides [74]. Hence, high expression levels of BCL-X_L_ exert cytoprotective effects along with the establishment of a state characterized by decreased levels of virtually all the metabolites involved in the Krebs cycle (but not of glycolytic substrates) as well as by reduced extents of N-*α*-acetylation [74]. Although Yi and colleagues ascribed such an antiapoptotic state (which could be reversed by the exogenous supply of citrate and acetate) only to the inhibition of N-*α*-acetylation [74], reduced levels of reactive oxygen species (ROS), which constitute a normal byproduct of mitochondrial respiration, may equally well underpin (at least part of) the cytoprotective effects that originate from BCL-X_L_ metabolic functions [75]. In support to this notion, (i) the overactivation of several metabolic circuitries (including glycogenolysis and glutaminolysis) and the overgeneration of ROS have been linked to both apoptotic and necrotic cell death [13, 76]; and (ii) a predominantly glycolytic metabolism, as observed in cancer cells even in the presence of normal oxygen levels (i.e., the so-called Warburg effect), reportedly exerts cytoprotective effects as it increases the amounts of reduced glutathione (a potent antioxidant) [75]. Of note, the pyruvate kinase M2, a glycolytic enzyme variant that is known to sustain the Warburg effect [77], has been shown to stimulate the expression of BCL-X_L_ at the transcriptional level [78]. Although the cytoprotective transcription factor NF-*κ*B may play a role in this setting [78], the precise molecular mechanisms underlying this phenomenon remain to be identified. In addition, protein acetylation has recently been involved in the regulation of autophagy (see below) [79–82], suggesting that BCL-X_L_ might exert a broad control over multiple cellular functions.

## 4. BCL-X_**L**_ and Autophagy

Autophagy is a catabolic pathway driving the lysosomal degradation of cellular constituents such as portions of the cytoplasm, protein aggregates, and dysfunctional/supernumerary organelles [83]. Under physiological conditions, autophagy plays a prominent role in the maintenance of intracellular homeostasis [5, 84, 85]. In addition, the autophagic flux is dramatically upregulated in response to a large panel of stress conditions, including (but not limited to) glucose and amino acid deprivation, hypoxia, intracellular pathogens, and cytotoxic xenobiotics [83, 86]. Although in some, mostly developmental, scenarios an autophagic program *de facto* mediates cell death [1, 7], stress-elicited autophagy near to invariably exerts prominent cytoprotective functions [87]. In line with this notion, both pharmacological and genetic maneuvers that block autophagy most often exacerbate, rather than limit, cell death as triggered by several distinct stimuli [88]. 

The stress-elicited upregulation of autophagy is a tightly regulated phenomenon, involving distinct molecular sensors and signal transduction cascades that impinge at various levels on the autophagic machinery. A detailed description of the proteins and factors that are involved in the regulation and execution of autophagy largely exceeds the scope of this paper and can be found elsewhere [83, 84, 87, 89]. Nevertheless, it is important to note that—in most (but not all) settings—autophagy critically relies on a class III phosphoinositide-3-kinase (PI3K) enzymatic activity [90]. In human cells, this function is mediated by phosphatidylinositol 3-kinase, catalytic subunit type 3 (PIK3C3, best known as hVPS34), which operates under the control of a multiprotein complex involving—among other interactors—the haploinsufficient oncosuppressor Beclin 1 [91, 92]. Importantly, by virtue of a *bona fide* BH3 domain [93], Beclin 1 can physically interact with antiapoptotic members of the BCL-2 protein family, including BCL-2 itself and BCL-X_L_ [94–96].

By interacting with Beclin 1, BCL-2 and BCL-X_L_ *de facto* prevent the stress-induced activation of autophagy [94]. In line with this notion, both “derepressor” BH3-only proteins (e.g., BAD) and chemical inhibitors of BCL-2-like proteins (e.g., ABT-737) have been shown to activate autophagy as they displace Beclin 1 from inhibitory liaisons with BCL-2 and BCL-X_L_ [94, 97]. Interestingly, some BH3-only proteins like BNIP3 have been shown to be critical for the execution of specific autophagic programs, such as the selective removal of damaged mitochondria (mitophagy) [5, 98], while their relevance in cell death regulation seems rather limited [99]. The binding of Beclin 1 to antiapoptotic BCL-2 family members can also be resolved by the phosphorylation of either binding partner [100–102]. Conversely, it seems that BCL-2 and BCL-X_L_ do not affect the steady-state levels of the autophagic flux in a direct fashion [100]. Of note, Tian et al. have recently suggested that BCL-2/BCL-X_L_-targeting compounds might activate a Beclin 1- and PIK3C3-independent autophagic program leading to cell death [103]. However, the authors failed to provide robust data to mechanistically explain their findings.

Besides a direct autophagy-modulatory function stemming from its interactions with Beclin 1 and other BCL-2-like proteins [94, 96], BCL-X_L_ is expected to regulate autophagy via less direct metabolic circuitries, notably as it (i) controls the efficiency of mitochondrial ATP production [67, 68], (ii) influences the exchange of critical bioenergetic metabolites (e.g., ATP and ADP) by PTPC components [24, 44], (iii) reduces the intracellular levels of acetyl-CoA [74], and (iv) interacts with the cytoplasmic pool of p53 [60, 65, 66]. Hence, the expression levels of BCL-X_L_ might also influence the autophagic flux in steady-state, as opposed to adaptive, conditions. This aspect of the crosstalk between BCL-X_L_ and autophagy warrants further investigation.

## 5. BCL-X_**L**_ and Other Cellular Functions

The implication of BCL-2 family members, including BCL-X_L_, in cell death-unrelated processes is not limited to the aspects of cell biology discussed above [28, 29]. For instance, it has recently been shown that BCL-X_L_ is phosphorylated at multiple serine residues (including S49 and S62) in a cell cycle-dependent fashion [104, 105]. The mitotic kinase Polo-like kinase 3 (PLK3) appears to be responsible for the cell cycle-dependent phosphorylation of BCL-X_L_ at S49 (starting at the S phase and abruptly falling at the onset of mitosis) [104], whereas PLK1 and mitogen-activated protein kinase 9 (MAPK9) have been suggested to catalyze BCL-X_L_ phosphorylation at S62 in response to DNA-damaging agents, hence stabilizing a cell cycle arrest at the G_2_ checkpoint [105]. Hence, similar to BCL-2 [106, 107], BCL-X_L_ plays a role in both physiological cell cycle progression and DNA damage-induced cell cycle checkpoints [104, 105]. 

A few years ago, rather unspecific inhibitors of BCL-2-like proteins such as ABT-737 and ABT-263 have generated an intense wave of enthusiasm and quickly entered clinical trials as part of antineoplastic regimens for the treatment of—mostly hematological—malignancies [108]. One of the most prominent on-target side effects of ABT-737 and ABT-263 turned out to be a dose-limiting thrombocytopenia [109, 110], linked to the fact that BCL-X_L_ is critical for the survival of platelets [111]. More recently, BCL-X_L_ has also been involved in the adhesive function of platelets [112, 113]. Hence, ABT-737 and ABT-263 appear to impair aggregation not only as they trigger the demise of a consistent fraction of circulating platelets but also as they exert consistent thrombocytopathic effects among residual platelets [113].

The implication of the BCL-2 protein family in mitochondrial dynamics as well as the actual relevance of mitochondrial fission/fusion events in apoptosis have been and still are the subject of a vivid debate [114–116]. While a detailed discussion of this topic largely exceeds the scope of this paper, it is worth noting that BCL- X_L_ has recently been shown to interact with (and stimulate the GTPase activity of) dynamin-related protein 1 (DRP1), a central component of the mitochondrial fission machinery [117]. In doing so, BCL-X_L_ appears to alter the mitochondrial function of neurons in a manner that stimulates the formation of synapses [117]. As DRP1 also participates in the execution of regulated necrosis [63], mitochondrial dynamics may constitute yet another point of control of cell death-related and -unrelated processes by BCL-X_L_.

BCL-2 and BCL-X_L_ have been reported to negatively regulate the NLRP1 inflammasome, a supramolecular platform that is required for the full-blown activation of caspase-1—and hence the production of interleukin (IL)-1*β* and IL-18—in response to proinflammatory stimuli [118, 119]. In particular, the flexible loop domain of both BCL-2 and BCL-X_L_ (which is located between the 1st and 2nd *α* helices of the proteins) appears to engage in physical interactions with NLRP1, thereby blocking the capacity of the latter to bind ATP and oligomerize [118, 120]. Besides playing a critical role in innate immunity, inflammasomes are crucial for the translation of immunogenic cell death (a functionally peculiar form of apoptosis) into a robust adaptive immune response [121]. It is therefore tempting to speculate, yet remains to be formally proved, that the interaction between antiapoptotic BCL-2 family members and inflammasomes may constitute a promising therapeutic target for enhancing the immunogenicity of (cancer) cell death [121].

## 6. Concluding Remarks

Following an initial wave of interest on the role of BCL-2-like proteins in the regulation of apoptosis, several laboratories have refocused their attention on distinct aspects of the biology of pro- and antiapoptotic members of the BCL-2 family [28, 29]. During the last decade, this intense investigational effort has led to the identification of several processes that are modulated by BCL-2 family proteins independent of (or at least not directly impacting on) their cell death-regulatory functions [28, 29]. As discussed in this paper, BCL-X_L_ has been shown to exert a consistent degree of control on various aspects of bioenergetic metabolism, including mitochondrial ATP production, Ca^2+^ fluxes, autophagy, and protein acetylation, as well as on several other cellular and organismal processes such as mitosis, platelet aggregation, and synaptic efficiency (Table 1). Hence, similar to other BCL-2 family members, BCL-X_L_ appears to operate at critical hubs to coordinately control multiple cellular functions including the three-step switch between homeostatic metabolisms, adaptive responses to stress, and cell death [122]. In this scenario, it is tempting to speculate, yet remains to be formally demonstrated, that BCL-2-like proteins may have originated as regulators of non-apoptotic functions and only later in evolution may have acquired the capacity of control cell death. Irrespective of this unresolved issue, we expect that—similar to what happened (and is still happening) for p53 [54]—the number of cellular functions that are regulated by BCL-2 family members, including BCL-X_L_, will grow further.

## Figures and Tables

**Table 1:** Functions of BCL-X_L_ at the interface between cell death regulation and other aspects of the cell biology.

Interactor	Main localization	Notes(s)	Reference
BAD	Cytosol mitochondria	By antagonizing BAD, BCL-X_L_ modulates the metabolic functions of a mitochondrial multiprotein complex involving glucokinase, PKA, and PP1	[64]
Beclin 1	Cytosol Golgi network	BCL-X_L_ binds to Beclin 1, thus inhibiting stress-induced, but not baseline, autophagy	[94, 97, 100]
DRP1	Mitochondria	BCL-X_L_ interacts with DRP1, altering the mitochondrial function of neurons to stimulate the formation of synapses	[117]
F_1_F_O_ ATP synthase	Mitochondria	BCL-X_L_ increases the enzymatic activity of the F_1_F_O_ ATP synthase, hence, stabilizing the Δ*ψ*_*m*_ and maximizing mitochondrial ATP synthesis	[67, 68]
IP_3_R	ER	BCL-X_L_ reduces Ca^2+^ concentration in the ER	[25–27]
Krebs's cycle	Mitochondria	BCL-X_L_ overexpression reduces the levels of virtually all TCA (but not glycolytic) intermediates, modulating both N-*α*-acetylation and autophagy	[74, 79–82]
MAPK9 PLK1	Cytosol nucleus	BCL-X_L_ is phosphorylated at S49 by PLK1 and MAPK9 in a cell cycle-dependent fashion	[104]
NLRP1 inflammasome	Cytosol	BCL-X_L_ inhibits the NLRP1 inflammasome, interfering with the secretion of IL-1*β* and IL-18	[118, 120]
p53	Cytosol mitochondria	BCL-X_L_ binds to p53, hence, inhibiting both its pro-apoptotic and metabolic functions	[54, 60, 66]
PKM2	Cytosol mitochondria	PKM2 stimulates the expression of BCL-X_L_ at the transcriptional level	[78]
PLK3	Cytosol nucleus	PLK3 phosphorylates BCL-X_L_ at S62 in response to DNA-damaging agents, favoring a cell cycle arrest at the G_2_ checkpoint	[105]
RAC2	Cytosol plasma membrane	In some settings, antiapoptotic BCL-2 family members exert prooxidant functions, perhaps linked to their interaction with RAC2	[70–72]
VDAC1	Mitochondria	BCL-X_L_ promotes the exchange of metabolites between the cytosol and the mitochondrial matrix	[24, 44]

Δ*ψ*_*m*_: mitochondrial transmembrane potential; DRP: dynamin-related protein 1; ER: endoplasmic reticulum; IL: interleukin; IP_3_R: inositol 1,4,5-triphosphate receptor; MAPK9: mitogen-activated protein kinase 9; PKA: protein kinase A; PKM2: pyruvate kinase M2; PLK: polo-like kinase 1; PP1: protein phosphatase 1; VDAC1: voltage-dependent anion channel 1.

## Abbreviations

AIF: Apoptosis-inducing factor
ANT: Adenine nucleotide translocase
APAF1: Apoptotic peptidase-activating factor 1
BH: BCL-2 homology
cFLIP: Cellular FLICE-inhibitory protein
cIAP: Cellular inhibitor of apoptosis protein
CYPD: Cyclophilin D
DCC: Deleted in colorectal carcinoma
DISC: Death-inducing signaling complex
DRP1: Dynamin-related protein 1
ENDOG: Endonuclease G
ER: Endoplasmic reticulum
FADD: FAS-associated protein with death domain
IL: Interleukin
IM: Mitochondrial inner membrane
IP_3_: Inositol 1,4,5-trisphosphate
MAPK9: Mitogen-activated protein kinase 9
MOMP: Mitochondrial outer membrane permeabilization
MPT: Mitochondrial permeability transition
OM: Mitochondrial outer membrane
PGAM5: Phosphoglycerate mutase family member 5
PI3K: Phosphoinositide-3-kinase
PI3KC3: Phosphatidylinositol 3-kinase, catalytic subunit type 3
PLK3: Polo-like kinase 3
PTPC: Permeability transition pore complex
PUMA: p53-upregulated modulator of apoptosis
RIPK1: Receptor-interacting protein kinase 1
ROS: Reactive oxygen species
TNFR1: Tumor necrosis factor receptor 1
VDAC: Voltage-dependent anion channel
Δ*ψ*_*m*_: Mitochondrial transmembrane potential.
